# Supplementary material for: Variation in the response to antibiotics and life-history across the major Pseudomonas aeruginosa clone type (mPact) panel
Source: Microbiol Spectr. 2024 Jun 11;12(7):e00143-24. doi: 10.1128/spectrum.00143-24 (PMC11218531; doi:10.1128/spectrum.00143-24)
Supplement: Supplemental material — Fig. S1-S9; Tables S1-S3. [file spectrum.00143-24-s0001.pdf]

Supplemental Material

Variation in the response to antibiotics and life-history across the major *Pseudomonas aeruginosa* clone type (mPact) panel

Authors:

Leif Tueffers[a,b]#, Aditi Batra[a,c], Johannes Zimmermann[a,c], João Botelho[a,c,d], Florian Buchholz[a], Junqi Liao[a], Nicolás Mendoza Mejía[e], Antje Munder[f,g], Jens Klockgether[f], Burkhard Tümmler[f,g], Jan Rupp[b,h], Hinrich Schulenburg[a,c]#

Affiliations:

[a] Evolutionary Ecology and Genetics, Zoological Institute, Kiel University, Kiel, Germany

[b] Department of Infectious Diseases and Microbiology, University of Lübeck, Lübeck, Germany

[c] Antibiotic resistance group, Max-Planck Institute for Evolutionary Biology, Ploen, Germany

[d] Centro de Biotecnología y Genómica de Plantas (CBGP), Universidad Politécnica de Madrid (UPM) - Instituto Nacional de Investigación y Tecnología Agraria y Alimentaria (INIA-CSIC), Madrid, Spain

[e] Institute of Clinical Molecular Biology, Kiel University, Kiel, Germany

[f] Department of Pediatric Pneumology, Allergology, and Neonatology, Hannover Medical School (MHH), Hannover, Germany

[g] Biomedical Research in Endstage and Obstructive Lung Disease Hannover (BREATH), German Center for Lung Research, Hannover, Germany

[h] German Center for Infection Research (DZIF), Hamburg-Lübeck-Borstel-Riems, Lübeck, Germany

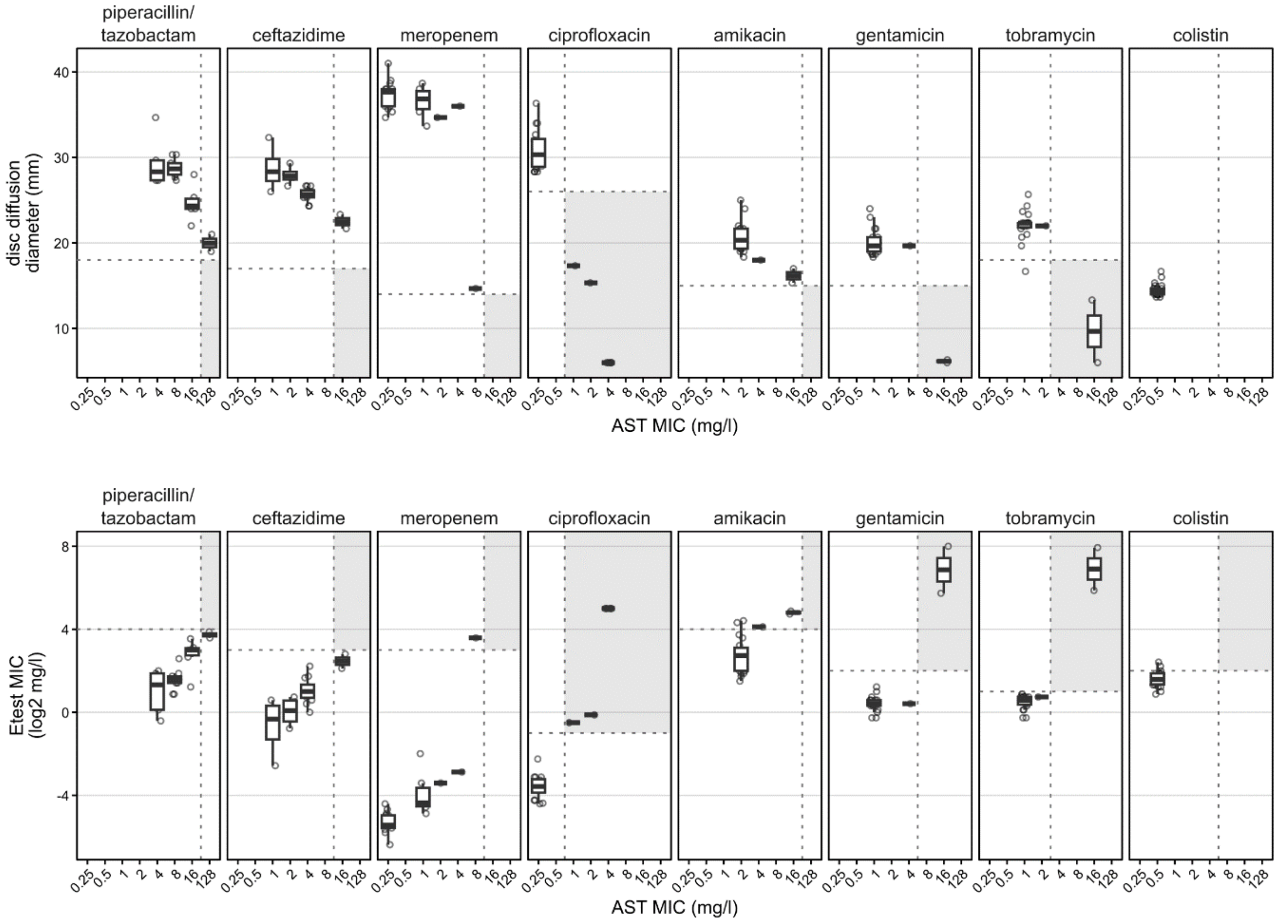

Figure S1: Comparison of automated susceptibility testing and M9 agar methods

Resistance breakpoints per method are indicated by dashed lines (AST vertical, M9 methods horizontal. Zones of categorical agreement of classification as ‘resistant’ are marked in gray.

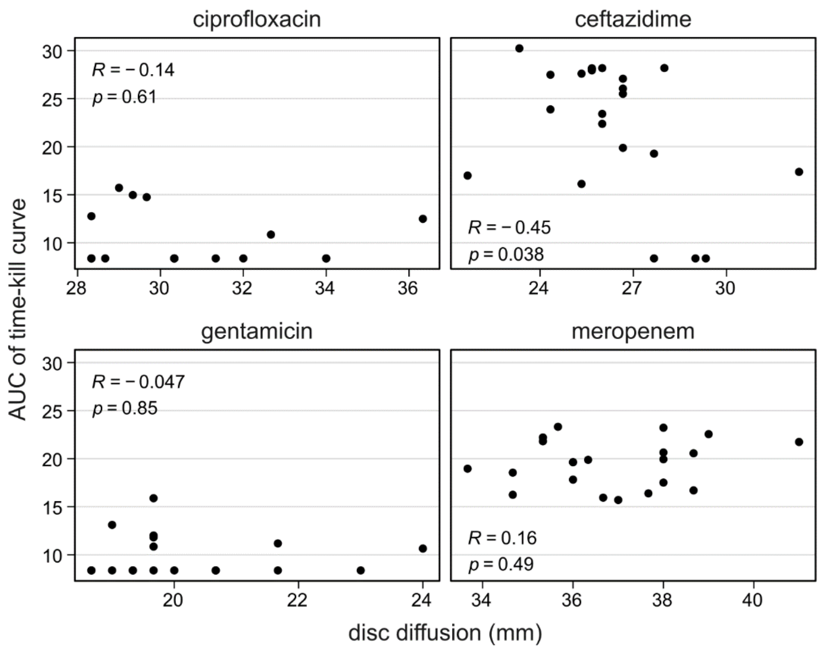

Figure S2: Correlation of strain MIC and area under the time-kill curve
Coefficient of correlation (R) and *p*-values of the t-test of *R* = 0.

Table S1: Modeled growth parameters

Strain	Growth rate (mean)	Growth rate (sd)	Lag phase (mean)	Lag phase (sd)	Maximum OD (mean)	Maximum OD (sd)	AUC (mean)	AUC (sd)
H01	0.0175	0.0053	121.51	52.31	0.27	0.03	157.84	19.55
H02	0.025	0.007	80.69	35.32	0.31	0.04	243.05	23.54
H03	0.0242	0.0049	63.18	27.93	0.25	0.02	185.23	22.2
H04	0.0258	0.0071	74.05	43.51	0.29	0.04	244.16	26.43
H05	0.0269	0.0058	61.01	20.47	0.33	0.02	261.26	9.38
H06	0.0206	0.0064	88.73	33.39	0.28	0.02	217.26	19.79
H07	0.0277	0.0057	49.65	20.54	0.35	0.05	285.66	25.95
H08	0.0237	0.0058	56.16	27.71	0.33	0.08	259.02	29.88
H09	0.0232	0.0044	58.92	16.81	0.31	0.03	257.41	33.68
H10	0.029	0.0036	62.69	13.55	0.41	0.06	268.85	21.24
H11	0.025	0.0068	45.72	9.98	0.33	0.05	275.44	28.74
H12	0.025	0.0072	43.29	18.82	0.34	0.05	283.25	26.85
H13	0.0249	0.0037	42.42	30.91	0.35	0.04	278.95	18.58
H14	0.0183	0.0056	74.76	42.92	0.36	0.04	294.11	23.02
H15	0.0155	0.0036	88.28	20.78	0.3	0.04	191.56	21.3
H16	0.0214	0.0042	67.84	24.05	0.34	0.04	267.23	11.79
H17	0.0235	0.0059	51.72	16.97	0.32	0.04	265.71	32.13
H18	0.0211	0.0047	77.01	30.51	0.34	0.04	262.06	19.73
H19	0.0228	0.0061	65.89	25.73	0.31	0.04	231.31	27.21
H20	0.0242	0.0057	57.03	20.78	0.31	0.02	251	29.15
PA14	0.0256	0.006	62.9	22.06	0.26	0.02	217.18	13.47
PAO1	0.0215	0.0035	57.59	20.12	0.28	0.02	249.8	9.75

AUC: area under the growth curve; sd: standard deviation. Lag phase given in minutes.

Table S2: Stationary phase colony forming units

Strain	cfu/ml	cfu/ml (sd)
H01	8,76E+08	1,43E+08
H02	1,39E+09	6,83E+08
H03	2,00E+09	5,61E+08
H04	1,09E+09	9,79E+08
H05	1,20E+09	2,49E+08
H06	1,12E+09	5,07E+07
H07	1,70E+09	4,02E+08
H08	1,49E+09	3,34E+08
H09	2,51E+09	3,19E+08
H10	2,82E+09	6,86E+08
H11	5,30E+09	8,64E+08
H12	5,17E+09	2,51E+08
H13	2,46E+09	4,33E+08
H14	3,26E+09	2,89E+08
H15	2,89E+09	3,75E+08
H16	2,80E+09	5,29E+08
H17	2,05E+09	1,21E+08
H18	2,12E+09	3,03E+07
H19	2,34E+09	5,46E+08
H20	2,59E+09	8,81E+08

cfu: colony forming units, sd: standard deviation of four biological replicates per strain. cfu/ml do not differ significantly (Kruskal-Wallis-test with 19 degrees of freedom, $p = 0,46$)

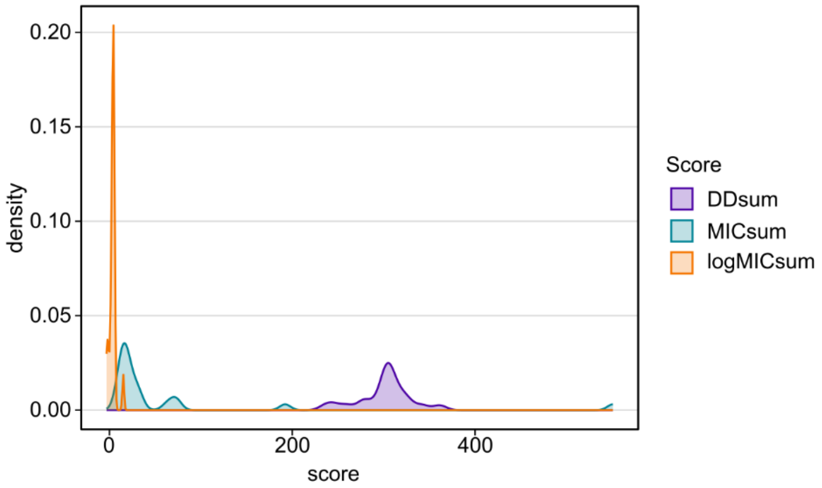

Figure S3: Density of strains’ cumulative resistance scores
Sum of disc diffusion diameters (DDsum), sum of gradient strip MICs (MICsum) and log2 transformed sum of differences between MICs and drug resistance breakpoints (logMICsum). Details see text.

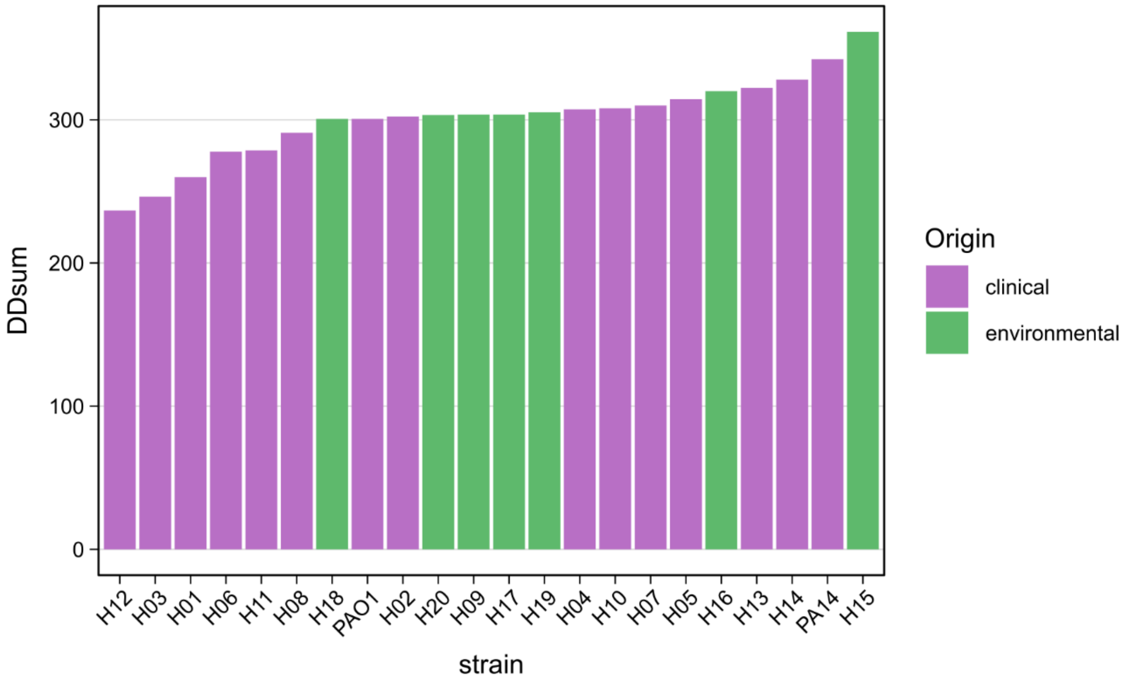

Figure S4: Strains sorted by cumulative resistance
Sum of disc diffusion diameters (DDsum) with strain origin marked as color.

Table S3: Statistical test overview

analysis	method	test statistic	fdr-corrected *p*
phylogroup A/B - logMICsum	Wilcoxon rank sum exact test	W = 44	1
phylogroup ABB - DDsum	Wilcoxon rank sum exact test	W = 41	1
origin - logMICsum	Wilcoxon rank sum exact test	W = 35	0.75
origin - DDsum	Wilcoxon rank sum test with continuity correction	W = 40	0.75
plasmids - logMICsum	Wilcoxon rank sum exact test	W = 2	0.94
plasmids - DDsum	Wilcoxon rank sum test with continuity correction	W = 5	1
logMICsum ~ ICEs	Pearson's product-moment correlation	t = 0.97	0.75
DDsum ~ ICEs	Pearson's product-moment correlation	t = -1.105	0.75
origin - growthrate	Wilcoxon rank sum test with continuity correction	W = 81.5	0.31
origin - lagphase	Wilcoxon rank sum exact test	W = 45	0.941
origin - auc	Wilcoxon rank sum exact test	W = 58	0.97
origin – maximum OD	Wilcoxon rank sum test with continuity correction	W = 51.5	1
phylogroup A/B - growthrate	Wilcoxon rank sum test with continuity correction	W = 29	0.75
phylogroup A/B - lagphase	Wilcoxon rank sum exact test	W = 41	1
phylogroup A/B - auc	Wilcoxon rank sum exact test	W = 54	0.75
phylogroup A/B – maximum OD	Wilcoxon rank sum test with continuity correction	W = 51.5	0.782
plasmids - growthrate	Wilcoxon rank sum test with continuity correction	W = 30	0.75
plasmids - lagphase	Wilcoxon rank sum exact test	W = 11	0.75
plasmids - auc	Wilcoxon rank sum exact test	W = 27	0.94
plasmids – maximum OD	Wilcoxon rank sum test with continuity correction	W = 22.5	1
ICEs ~ growthrate	Pearson's product-moment correlation	t = -0.96	0.75
**ICEs ~ lagphase**	**Pearson's product-moment correlation**	**t = 3.366**	**0.044**
**ICEs ~ auc**	**Pearson's product-moment correlation**	**t = -3.6**	**0.04**
ICEs ~ maximum OD	Pearson's product-moment correlation	t = -2.095	0.309

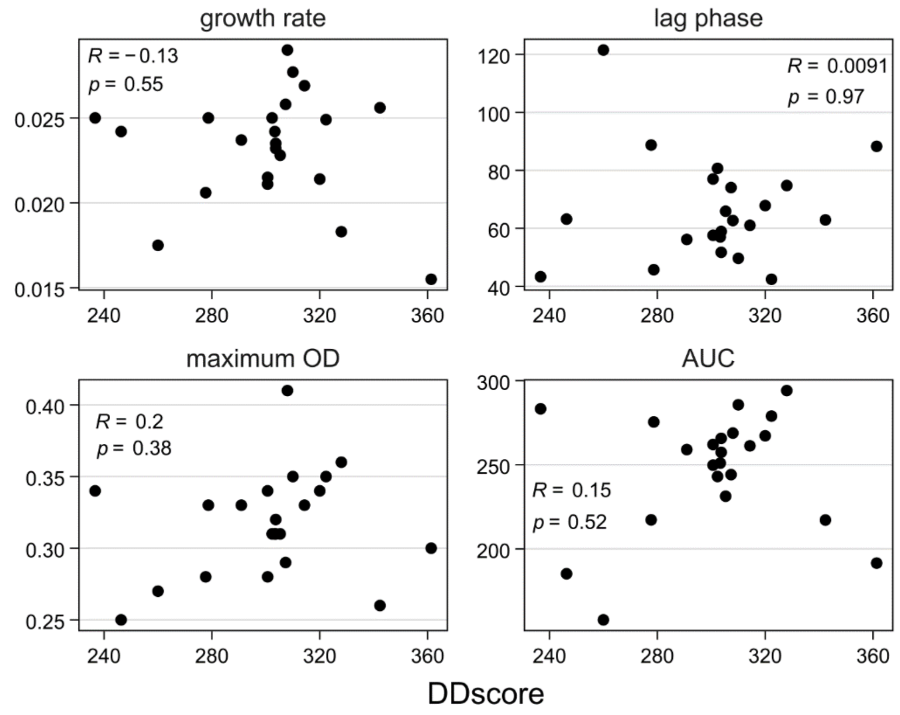

Figure S5: Correlation of growth parameters and resistance
The sum of disc diffusion diameters for 12 drugs (DDScore) is correlated with the modeled growth parameters of the individual strains (coefficient of correlation R and t-test for *R* = 0 as *p*)

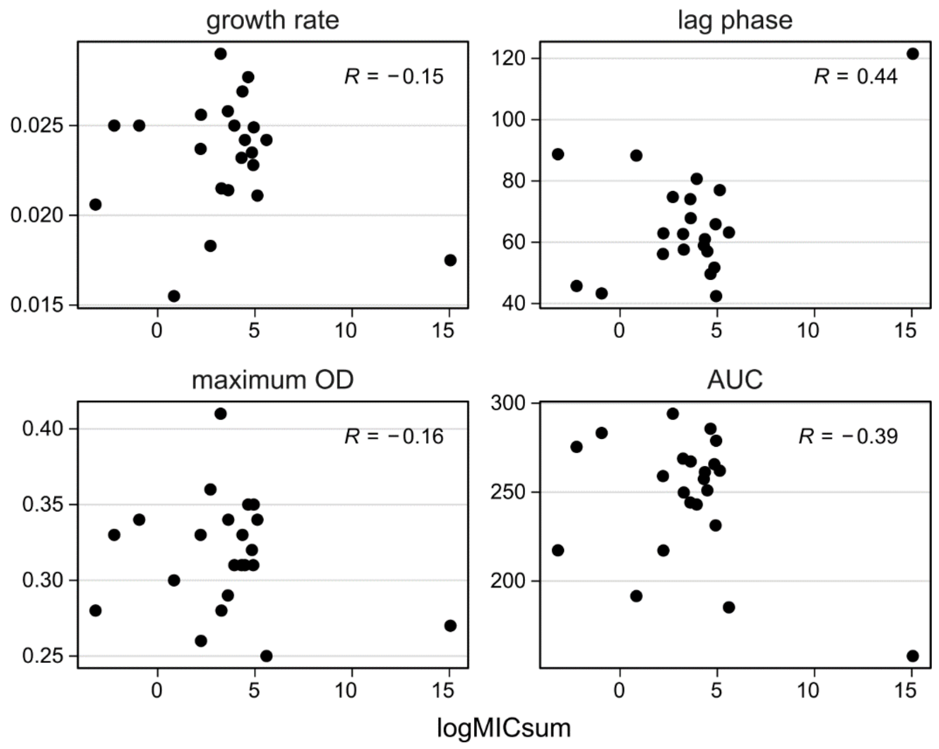

Figure S6: Correlation of growth parameters and gradient strip resistance
The log2 transformed sum of differences between MICs and drug resistance breakpoints (logMICsum) is correlated with the modeled growth parameters of the individual strains.

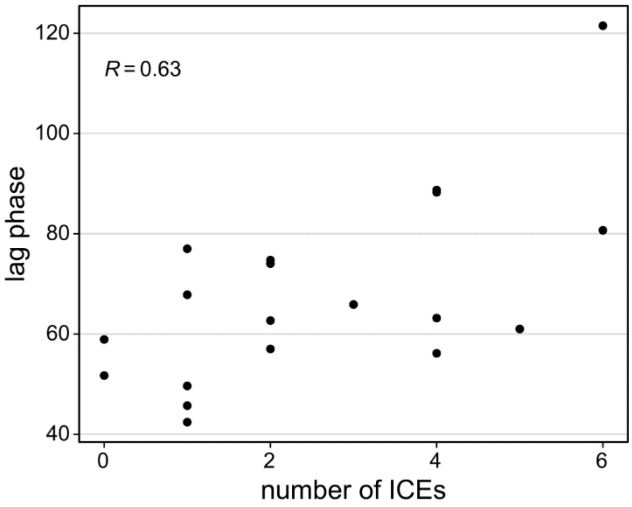

Figure S7: Correlation of lag phase and number of integrative conjugative elements per strain

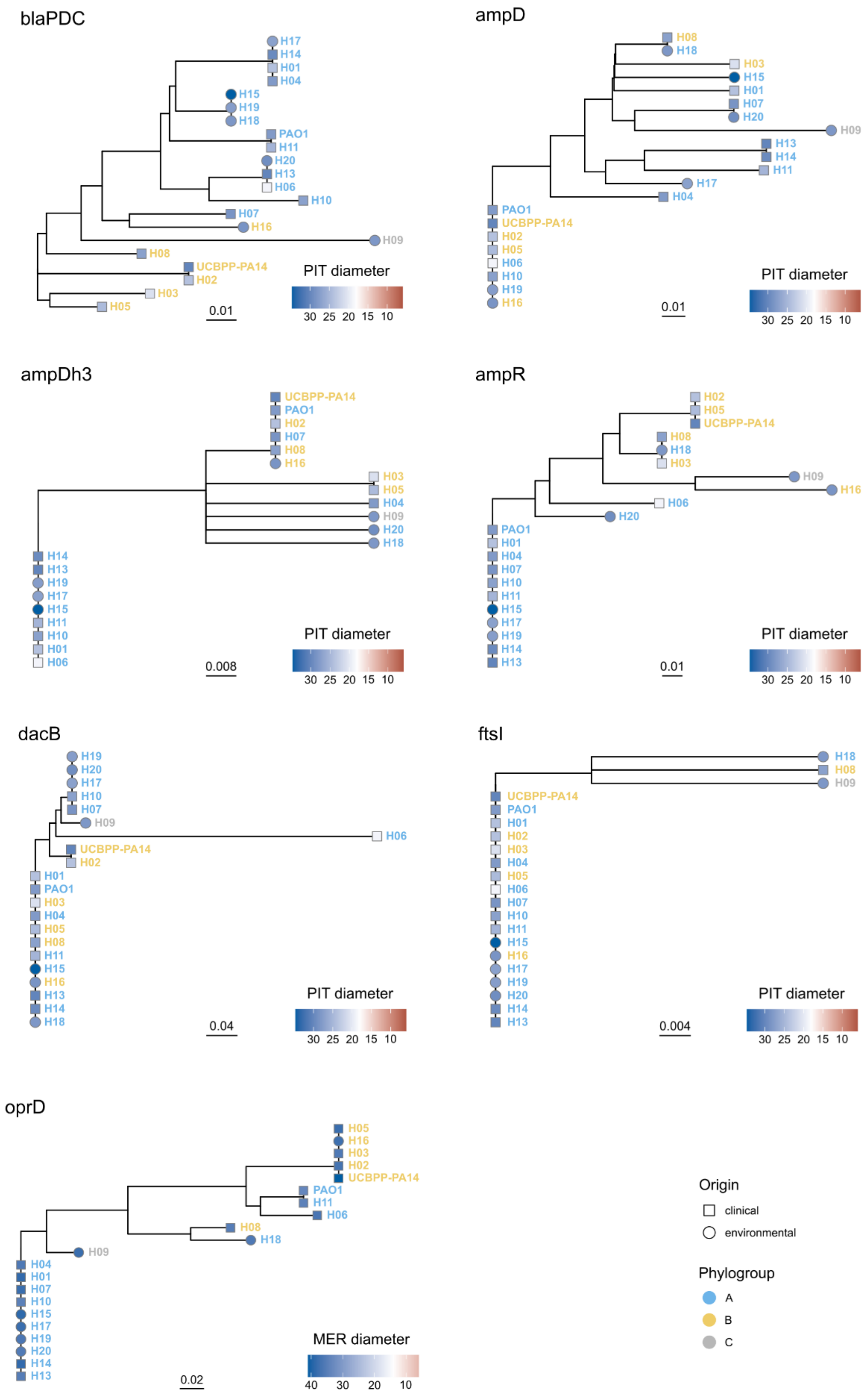

Figure S8: Betalactam resistance gene phylogenies

Neighbor-joining trees of amino acid sequences of the indicated genes. Strain names at the edges are shaded by phylogroup and shaped by strain origin. Resistance to an indicator drug (PIT = piperacillin/tazobactam, MER = meropenem) is given as the color of the edges, with the values centered around the resistance breakpoints in white and values below in blue.

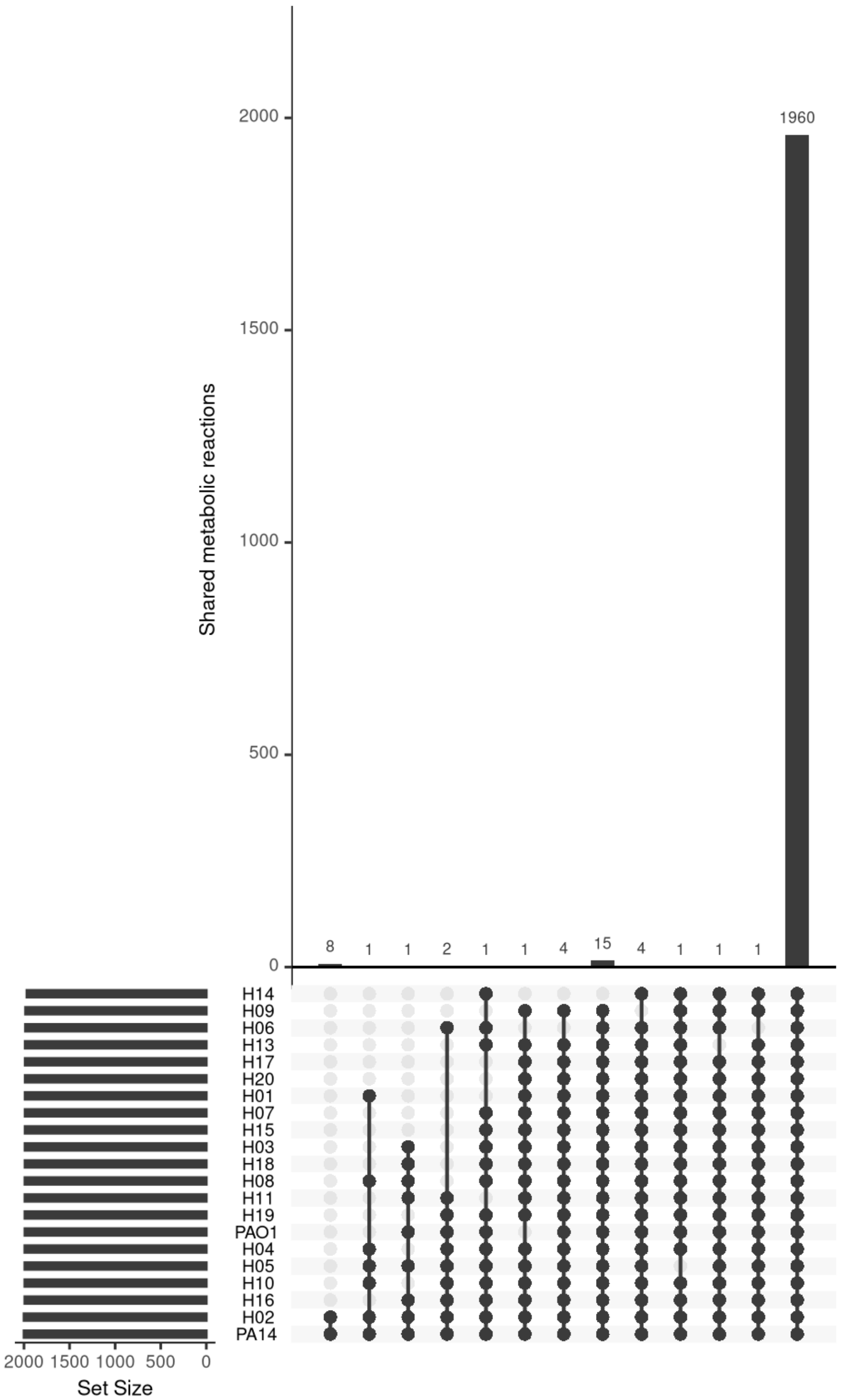

Figure S9: Shared metabolic reactions

Comparison of the occurrence of metabolic reactions between metabolic models of the mPact strains. The upset plot shows intersections of shared reactions.
